# The perceived quality of video consultations in geriatric outpatient care by early adopters

**DOI:** 10.1007/s41999-022-00678-6

**Published:** 2022-08-13

**Authors:** Romy Spronk, Hester J. van der Zaag-Loonen, Nicole Bottenberg-Wigbold, Nadine Bovee, Rosalinde Smits, Marjolein van Offenbeek, Janita F. J. Vos, Marie Louise Luttik, Barbara C. van Munster

**Affiliations:** 1 University of Groningen, University Center for Geriatric Medicine, University Medical Center Groningen, PO Box 30.001, HPC AA-43, Groningen, The Netherlands; 2 Faculty of Economics and Business, University of Groningen, Groningen, The Netherlands; 3 Academy of Nursing, Hanzehogeschool Groningen, Groningen, The Netherlands; 4 Geriatrics Department, Leiden University Medical Center, Leiden, The Netherlands

**Keywords:** Geriatrics, Tele-health, Covid-19, Qualitative research, Thematic analysis

## Abstract

**Purpose:**

The COVID-19 pandemic caused rapid implementation and upscaling of video consulting. This study examined the perceived quality of care delivered through video consulting at a geriatric outpatient clinic, and how this related to adoption issues and barriers early adopting professionals found themselves confronted with.

**Methods:**

We performed a qualitative study using semi-structured interviews with healthcare professionals complemented by the views of geriatric patients, family caregivers and medical secretaries. Participants from five academic centers and six teaching hospitals were included. Three researchers conducted the interviews, coded the data, and used thematic analysis.

**Results:**

Interviews were conducted with 13 healthcare professionals, 8 patients, 7 family caregivers, and 4 medical secretaries. From these early adopters, we infer five criteria positively contributing to perceived quality of care provided by video consulting: (1) the patient has an intact cognitive function; (2) a family caregiver with digital literacy can be present; (3) doctor and patient already have an established relationship; (4) no immediate need for physical examination or intervention; and (5) the prior availability of a comprehensive and concise medical history. Overall, the uptake of video consulting in geriatric outpatient care appeared to be slow and laborious due to several implementation barriers.

**Conclusion:**

The implementation of video consulting use among geriatricians and geriatric patients at the geriatric outpatient clinic was slow due to the absence of many facilitating factors, but video consulting might be offered as an alternative to face-to-face follow-up to suitable patients in geriatric outpatient clinics.

**Supplementary Information:**

The online version contains supplementary material available at 10.1007/s41999-022-00678-6.

## Key summary points

**Aim:**

To enquire the perceived quality of care delivered through VC at a geriatric outpatient clinic from a healthcare professional’s perspective triangulated with the views of others.

**Findings:**

The implementation of video consulting at the geriatric outpatient clinic was slow due to the absence of many facilitating factors, but participants believe video consulting can be of future use for particular geriatric patients. Both efficiency and comfort gains and losses were mentioned for and by healthcare professionals and patients.

**Message:**

In the geriatric population, consideration should be given to the cognitive functioning of the patient and the presence of a digitally literate person when adopting video consultations.

**Supplementary Information:**

The online version contains supplementary material available at 10.1007/s41999-022-00678-6.

## Introduction

The COVID-19 pandemic caused rapid implementation and upscaling of forms of telemedicine (also referred to as telehealth), in particular to protect vulnerable patients from being infected during a hospital visit. Videoconsulting (VC) provides a medium to improve access to care and may contribute to health, wealth and efficiency gain strategies [1]. Studies assessing experiences of VC use in hospitals with older patients, further referred to as geriatric patients, are scarce. Available studies show that on the one hand, VC support health practice, especially as a useful alternative when face-to-face consultations (FtFC) are not possible [2]. On the other hand, VC can be challenging for geriatric patients as they have limited familiarity with the technology and may experience sensory or cognitive impairments [3, 4]. Additionally, the consultation delivered at the geriatric outpatient clinic is comprehensive from a broad multidisciplinary perspective and many functional tests are involved, challenging a full-fledged VC.

Geriatric patients are generally but not exclusively 70 years or over. However, other factors than chronological age define the geriatric patient, such as functional, cognitive and social problems and the presence of multimorbidity [5]. HCPs usually estimate patients’ frailty by assessing their physical and mental state and their dependence on others in daily activities [6]. Van Houwelingen et al. showed how older adult’s willingness to use VC depended on their perceived privacy and security, self-efficacy, expectations about VC performance and the efforts required by them [7]. Importantly, however, these preliminary findings on the technical and practical feasibility of VC use for geriatric patients, leave the question as to how they and their HCPs perceive the quality of care through VC, unanswered.

Studies on HCPs’ perceptions about VC use with other than geriatric patients, show contradictory results. HCPs evaluated VC as a safe substitution for FtFCs in a palliative home care setting [8]. HCPs valued VC as it improved their ability to deliver synchronized care and it improved the communication and quality of care due to increased co-operation between HCPs [8]. However, in an orthopedic rehabilitation setting, some HCPs appointed their reduced capacity to be flexible in time due to their high workload, for example, disabling them to support patients with VC technology [9]. Moreover, VC technology implementations appeared more complex and time-consuming than expected and barely provided HCPs the expected costs- or time savings [10]. Finally, in a colorectal oncological care setting, HCPs foresaw difficulties in shaping a complete patient picture when physical contact is absent [11].

The aim of this study, therefore, was to explore the perceived quality of care by HCPs when delivering care during the start-up phase of VC at a geriatric outpatient clinic, and how this related to adoption issues and barriers early adopters found themselves confronted with. We complemented HCPs experiences with those of patients, caregivers and medical secretaries to arrive at a rich understanding of the first experiences with VC in geriatric outpatient care.

## Methods

A qualitative study was performed between October 2020 and February 2021 to create an in depth understanding of the nature of the phenomenon of interest, using thematic analysis [12]. Our approach relied on combining existing theoretical models with new empirical data to incrementally build more powerful models. The Consolidated Criteria for Reporting Qualitative research (COREQ) checklist was followed [13]. Eleven Dutch hospitals were involved in this study, including six university medical centers and five regional hospitals. All hospitals started VC as a substitution for hospital-based appointments during the SARS-COVID19 pandemic and were evenly involved in this study.

### Recruitment

HCPs were approached both through a national survey and personally. The national survey explored the extent to which VC was being implemented in the geriatric population, and identified those hospitals that would be able to provide participants. HCP from these hospitals working in the field of geriatric medicine were contacted and selected by convenience sampling.

We added a convenience sample of geriatric patients, family caregivers and medical secretaries to complement the HCP’s view. Patients were eligible to participate if they were treated at the geriatrics outpatient clinic and had recently experienced a VC, which could be a first time or a follow-up visit. Inclusion criteria involved adequate speaking and hearing skills. The family caregiver(s) of the included patient were eligible to participate if they had been involved in one or more VCs with the patient. The involvement of a family caregiver during the consultation is almost standard practice at geriatric outpatient clinics, as well as an extended duration of the consult compared to outpatient clinics of other medical specialties. Care processes at the geriatric outpatient clinic consist of diverse examinations in a multidisciplinary team (including cognition test, fall risk analysis, and comprehensive screening for high-risk treatment) and are characterized by shared decision-making. Medical secretaries were responsible for the administrative handling of VC consultations at the geriatric outpatient clinic. We included secretaries to better assess the scope of their involvement and how it might impact any patient pre-selection and support, and thereby perceived quality of care. All participants were unknown to the interviewer.

### Data collection process

Data were collected using semi-structured interviews. Three of the authors (RS, NW, NB) conducted the interviews through video calling. These video sessions were audio recorded. The HCP-interview guide contained three main areas: personal details, perceived quality of care, and implementation details.

Personal details served to collect information on prior experience with video calling. Questions on the perceived quality of care were based on the six domains of ‘Good Quality Care’: safety, effectiveness, patient-centeredness, timeliness, efficiency and equivalence [14] (see Table 1). Questions on implementation details served to examine digital literacy, independency during VC, general views and preferences and expectations for future outpatient follow-up by means of VC. A concise summary of each interview was sent to and verified by all participants to apply member checking [15]. The interview guide was evaluated and adapted regularly by the research team following an iterative process. The final interview guide is shown in Online Appendix A. We aimed to collect data following the data saturation guidelines by Morse, meaning we continued our process of interviewing until no new elements of discourse were collected per participant group [16].

**Table 1:** The six domains of Good Quality Care

Domain	Definition
Safety	To avoid harm to patients from the care that is intended to help them
Effectiveness	To provide services based on scientific knowledge to all who could benefit and refraining from providing services to those not likely to benefit
Patient-centeredness	To provide care that is respectful of and responsive to individual patient preferences, needs, and values and ensuring that patient values guide all clinical decisions
Timeliness	To reduce waits and sometimes harmful delays for both those who receive and those who give care
Efficiency	To avoid waste, including waste of equipment, supplies, ideas, and energy
Equivalence	To provide care that does not vary in quality because of personal characteristics such as gender, ethnicity, geographic location, and socioeconomic status

[14] Institute of Medicine (US) Committee on Quality of Health Care in America. Crossing the Quality Chasm: A New Health System for the 21st Century. Washington (DC): National Academies Press (US); 2001

### Data analysis

The interviews were transcribed verbatim and then analyzed using *ATLAS.ti software (version 8)*. Interviews and analysis occurred concurrently.

For thematic analysis, the approach as outlined by Braun and Clarke was applied, which consists of six phases [17]. Deductively, the initial coding frame was based on the six quality-of-care domains and thereby provided themes. Inductively, the coding frame was revised iteratively when other (sub)-categories emerged. Notably, in, especially the HCP data, implementation issues continuously popped up in relation to the interviewees’ reflections on the quality of care provided. Therefore, we took these along as extra categories in our analysis. Subcategories were developed for larger themes to support precise data analysis. For example, theme 2: effectiveness of care, was divided into 6 subcategories because of its wide scope. The three authors who conducted the interviews consequently first independently coded the data and second commonly discussed the definition and (sub)-categorization of codes throughout the cyclical coding process to ensure accurate representation of the data. The final codebook is shown in Online Appendix B. An audit trail was kept during the coding process to increase the reliability of our findings [15].

After the coding, the data were analyzed and then compared per interviewee subgroup to explore and understand the differences and similarities in perspectives and experiences. Reflexivity was attempted through continuous critical questions about the interpretations and views of the data analysist by the multidisciplinary team of co-authors during the whole research process.

## Results

The final sample consisted of 13 HCPs (11 geriatricians, 1 physician assistant and 1 nurse specialist), 8 patients, 7 family caregivers, and 4 medical secretaries. Characteristics of the interviewees are shown in Table 2. Data saturation only occurred for HCPs, not for the other participant groups (patients, family care givers and medical secretaries). In line with our research aim, we present the findings per subquestion concerning: 1) the perceived quality of care and 2) implementation issues.

**Table 2:** Interviewees characteristics

Healthcare professional code	Triad number	Function	Duration of interview (minutes)	Gender	Age	Previous personal experience with video calling
HCP01	–	Geriatrician	30:36	Male	36–45 years	0–1 year
HCP02	–	Geriatrician	28:31	Female	36–45 years	0–1 year
HCP03	–	Physician	41:27	Female	26–35 years	> 10 years
HCP04	–	Physician	32:19	Female	26–35 years	> 5 years
HCP05	–	Geriatrician	29:56	Female	36–45 years	> 10 years
HCP06	–	Geriatrician	38:21	Female	46–55 years	> 5 years
HCP07	–	Geriatrician	36:45	Male	46–55 years	0–1 years
HCP8	1	Physician	45:05	Female	36–45 years	0–1 year
HCP9	2/3/4	Physician	52:22	Female	46–55 years	0–1 year
HCP10	5	Physician	34:36	Female	56–65 years	> 10 years
HCP11	6	Physician	44:54	Female	36–45 years	> 10 years
HCP12	7	Physician assistant	38:01	Male	46–55 years	0–1 year
HCP13	8	Nurse specialist	33:26	Female	36–45 years	0–1 year

Medical secretaries				
Medical secretary code	Function	Duration of interview (minutes)	Gender	Age
MES1	Medical secretary	26:25	Female	36–45 years
MES2	Medical secretary	24:07	Female	56–65 years
MES3	Medical secretary	25:32	Female	26–35 years
MES4	Medical secretary	15:58	Female	36–45 years

Patients					
Patient code	Triad number	Duration of interview (minutes)	Gender	Age	Previous experience with video calling
P01	1	42:00	Male	82	No/limited
P02	2	23:00	Male	75	No
P03	3	28:45	Female	87	No/limited
P04	4	30:23	Male	73	Yes
P05	5	39:21	Female	74	No
P06	6	32:35	Male	56	Yes
P07	7	42:27	Female	76	No
P08	8	51:03	Female	79	No/limited

Family caregivers					
Family caregivers code	Triad number	Duration of interview (minutes)	Gender	Age	Previous experience with video calling
FC01	1	35:32	Female	58	Yes
FC02	1	31:05	Male	82	Limited/yes
FC03	2	44:49	Female	73	No
FC04	3	37:08	Female	82	Yes
FC05	5	33:33	Male	50	Yes
FC06	7	53:16	Male	51	Yes
FC07	8	31:13	Male	80	Yes

## Perceived quality of care of video consulting

### Safety of care

HCPs addressed privacy as an important factor related to this theme. First, HCPs told they preferred to work in a quiet and enclosed working space to maintain the confidentiality of the conversation; which was a challenge when working from home. The majority of patients and family caregivers stated they did not worry about privacy because they trusted the procedures of the hospital.

The absence of physical examination was expressed to be a major disadvantage of VC use. This absence caused insecurities among HCPs about the safety, effectiveness, completeness and carefulness of their consult and consequently the risks of misdiagnoses. Some HCPs, however, invented creative ways to execute part of the physical examination, such as to verbally instruct patients to do simple walking exercises and cognitive tests through VC. Some of these were considered to be adequate.

### Effectiveness of care

Among this theme, three subthemes were identified: positive attitude of user, interaction during VC, and role of family caregiver.

*Attitude of user—*The predominant attitude toward VC use among HCPs was positive. Many HCPs were willing to discover the possibilities of VC, however, feelings of apprehension were repeatedly mentioned as part of the start-up phase of implementing VC. Patients and family caregivers also predominantly had an open and willing attitude toward VC use.

*Interaction during VC—*The interaction between HCP and patient was perceived as comparable compared to a FtFC, but many expressed it to be substantially better than during a telephone consultation (TC). Some HCPs stressed they preferred the interaction during a FtFC compared to VC. HCPs emphasized the importance of visual contact: it enabled non-verbal communication which helped them to understand the patient’s condition and to show the interaction between patient and family caregiver. However, one HCP stated,

“I think that this type of patient group and their problems is too complex, so video calling as a medium is insufficient to properly draw a complete patient picture. We cannot even draw this picture when they sit in front of us. Let alone if you have to do it by a video call.” (HCP1)

*Role of family caregiver*—VC positively contributed to gathering the medical history, as it enabled the online participation of family caregivers. Almost all of the included patients were dependent on their family caregiver for technical VC preparation. Family caregivers functioned as an “interpreter” between HCP and patient.

“I think it is a good thing because I understand the doctor better than she does and also better than my father, it is still a certain language that doctors speak.” (FC1)

### Patient-centeredness of care

Patients reported they were happy they could receive care without having to travel to the hospital during the pandemic. HCPs also emphasized that a VC appointment could contribute to the comfort of a patient and their family caregiver, as a hospital visit is often perceived as exhausting and time-consuming. This advantage of VC appointments was thought to increase as the vulnerability of patients increases as well. HCPs also mentioned the stress levels of patients, which could be both negatively and positively influenced by VC appointments. On the one hand, VC could decrease stress levels as patients could stay at home. On the other hand, VC could increase stress levels through technical unfamiliarity.

### Efficiency of care

In general, the required preparation time for HCPs for a VC was short and it was perceived to convenient when they had an existing relationship with the patient. The total duration of a VC appointment was perceived as shorter compared to a physical consult. However, some HCPs took more time for the medical history because it took longer to obtain all the relevant information. In case a follow-up consultation was necessary for physical examination at the hospital, both efficiency advantages and disadvantages were mentioned.

“But it will lead to patients being here for half an hour instead of an hour when they return. That saves 30 min of exposure to potential danger.”(HCP3)

and

“First you have a video consult of 1,5 hours and then they come by again for 1,5 hours. In that respect it is not more efficient.” (MES3)

### Equivalence

HCPs generally felt that they could adequately provide care to a wide variety of geriatric patients through VC use. However, for some patients, HCPs considered an FtFC as the best way to make a physical and cognitive patient assessment, mainly because it was perceived to be more personal and because they could better observe patient’s physical responses as well as the interaction between patients and family caregivers.

### Boundary conditions for VC use (inductive category within perceived quality of care)

HCPs outlined a number of boundary conditions for effective VC use. The importance of an existing doctor-patient relationship was frequently mentioned, leading to a higher suitability of VC use for follow-up versus first consultations. The consult content was also mentioned by HCPs: bad news and complicated conversations were not suitable for VC, as well as consults which required a physical examination. On the contrary, both patient and informant history were perceived as very suitable to be executed by VC. Finally, patient characteristics such as a adequate level of cognitive functioning, good hearing and little frailty were also repeatedly emphasized.

## VC implementation issues

The themes that were raised included technical support, VC technical performance, users’ digital literacy, adoption rate, and future improvements of VC use.

*Technical support*—Help with technical support and VC equipment from the hospital was perceived differently by the interviewed HCPs. Staff training during the implementation phase, purchased specialized hardware, and collaborations with internal or external agencies that could be contacted for technical support all contributed positively to HCP’s willingness to use VC. However, sufficient VC equipment was frequently lacking, and HCPs said they did not experience support and expressed frustration about this.

*VC technical performance*—HCPs experienced many technical issues. In some cases, they switched from VC to a TC because of these technical problems. Lack of image or sound, a reverberating sound, or a suboptimal internet connection were frequently mentioned. Patients and family caregivers also experienced such technical issues and mentioned these as a major disadvantage of VC use.

Also, the workload of a VC appointment as experienced by HCPs was perceived higher during the start-up phase and in those hospitals where the VC software had not been integrated into the electronic patient record system. The latter caused additional steps that had to be taken to start the VC appointment, negatively influencing efficiency. During the start-up phase, technical unfamiliarity and technical issues lead to a higher workload among HCPs.

*Users’ digital literacy*—Initially, the digital literacy of some HCPs was limited, but this improved during the adoption process. Limited digital literacy of patients and family caregivers was frequently mentioned by HCPs as a restricting factor for VC use. However, some HCPs also reported they were positively surprised by the digital literacy and the technical equipment of patients.

“Quite often things went well for this target group. That is, I thought that was a nice step. So older adults who still found a phone or iPad somewhere, and got that thing working, yes.” (HCP7)

*Adoption rate—*A slow adoption rate was observed at the geriatrics department. HCPs attributed this to the complexity of the geriatric care process. Many patient examinations in geriatrics are not commonly executed by VC due to the necessity of physical contact. Examples include fall risk examination and cognitive tests.

*Improvements of VC use—*HCPs agreed that VC use will increase in the future, as older adult patients will also become more technically skilled over time and the apprehension will decrease. Patients suggested that it would be helpful to be more informed during the waiting process: a confirmation that the doctor is aware of the patient’s presence and an estimate of the remaining waiting time would reduce the feeling of insecurity among patients.

## Discussion

The aim of our study was to examine the perceived quality of care delivered through video consulting at a geriatric outpatient clinic, and how this related to adoption issues and barriers early adopting professionals found themselves confronted with. The results from this study indicate that VC implementation was a slow and laborious process within this medical discipline in the included hospitals in our country, mainly due to lacking facilitating support from the hospital, assumptions about the digital literacy of geriatric patients, and the complexity of the geriatric care process including many diagnostic methods requiring physical attendance. However, HCPs agree that VC use has several advantages primarily for patients and family caregivers, as they save a generally uncomfortable traveling and waiting process for a hospital visit for them, and family members can join the consultation without being physically present. For HCPs themselves, VC use could provide an organizational efficiency advantage compared to FtFCs, but not for all patients. Compared to telephone consultations, VC use provides HCPs with valuable additional non-verbal patient-specific information.

The results from our study confirm previously identified barriers to VC use, such as low self-efficacy and low digital literacy in patients, as outlined by Van Houwelingen et al. [7], and diminished cognition among some geriatric patients, as patients with a low cognitive function were more difficult to connect to and interact with. Van Houwelingen et al. also mentioned perceived privacy and security as predictive factors for VC use, but these factors did not play a determining role among our participants [7]. On the other hand, the lack of a supportive infrastructure, as mentioned by Seiffert et al. [18], was also the main barrier for implementation as mentioned by our participants, which is confirmed by the study of Haydon et al. who describe recommendations regarding infrastructure for adoption of VC use in geriatric medicine.[19]. While COVID-19 served as a catalyst for the adoption of telemedicine in some geriatric clinical settings [20], this was less so among our geriatric outpatient setting.

We encountered a contradiction during our study. HCPs mentioned that on the one hand, VC was”insufficient to properly draw a complete patient picture” in geriatric patients, which has also been emphasized by Barsom et al. [11]. On the other hand, many confirmed that a VC was more convenient for geriatric patients as it saves them an uncomfortable and time-consuming travel process [11, 20]. An accurate assessment of all different perspectives has to be made on the additional value of VC use compared to a physical consult. The geriatric assessment of the patient’s level of frailty should play a determining role in this assessment. This contradiction emphasizes again the importance of customization of VC use among geriatric patients, partly due to the many and heterogenous factors underlying frailty in geriatric patients.

Our findings should be interpreted in the context of a few limitations. First, our sample size of patients and family caregivers was restricted due to slow adoption of TC at geriatric outpatient clinics, and subsequently maximum variation sampling was not possible for HCPs, and data saturation did not occur for patients and family members. This lead to including mainly early-adopters of VC, which does not comply with maximum variation sampling as would be preferred in qualitative research. The results therefore may be more in favor of VC use than would be obtained in late-adopters. Strengths of our study include our multidisciplinary research team, the triangulation of perspectives through both HCPs, patients, caregivers and medical secretaries, and the fact that we were one of the first groups to examine VC use during the pandemic in this clinical setting.

It is important to emphasize the likely influence of the Covid-19 pandemic on our study results. Results of a study by Eberly et al. suggested that the Covid-19 pandemic increased the digital divide, which is defined as the gap between those who have and do not have access to computers and the Internet [22]. Covid-19 might in this way impede access to eHealth services, such as VC, for disadvantaged population groups, such as geriatric patients [21]. Several recent studies addressed the negative influence of Covid-19 on the digital divide [21–24]. Based on our study results, we expect that Covid-19 might stimulate digital literacy among geriatric patients as many started to use digital devices to stay connected to their relatives. We observed a predominantly willing and positive attitude among geriatric patients toward VC use, both for hospital-related and personal use.

A major contribution of this study is that it shows the importance of customization of VC use in geriatric patients. In contrast to the study by Barsom et al. among patients with colorectal cancer, perceived quality of care through a VC was not always found to be equal compared to a physical consult [11]. More specifically, a lack of digital literacy among geriatric patients frequently led to technical issues impeding the visual and auditory contact during the call. Nevertheless, HCPs emphasized how the digital literacy of patients and their family caregivers frequently surprised them. Therefore, in geriatric outpatient clinics, the choice between FtFC and VC is not a matter of ‘one size fits all’ and customization of VC use is necessary. HCPs’ perceived quality of care using video consulting at geriatric outpatient clinics was patient-specific, which accords with the other stakeholders’ experiences. Both efficiency gains and losses were mentioned when compared to FtFCs. The following criteria positively related to the perceived quality of care: (1) the patient has an intact cognitive function; (2) a family caregiver with digital literacy can be present; (3) doctor and patient already have an established relationship; (4) there is no immediate need for physical examination or intervention; and (5) a comprehensive and concise history has already been performed. Sufficient technical support is a prerequisite for fast implementation. We suggest using these as selection criteria for choosing which patients can be offered a VC instead of a FtFC. Obviously, a general precondition for VC is a stable, smooth-running VC environment that the HCPs and patients have familiarized themselves with. We encountered willingness to use VC at the included geriatric outpatient clinics and low adoption at the same time.

## Supplementary Information

Below is the link to the electronic supplementary material.

Supplementary file1 (DOCX 27 kb)
