# Supplementary material for: The perceived quality of video consultations in geriatric outpatient care by early adopters
Source: Eur Geriatr Med. 2022 Aug 13;13(5):1169–76. doi: 10.1007/s41999-022-00678-6 (PMC9376038; doi:10.1007/s41999-022-00678-6)
Supplement: Supplementary file 1 — Supplementary file1 (DOCX 27 kb) [file 41999_2022_678_MOESM1_ESM.docx]

# **Supplementary files**

1. **Interview guide HCPs**

***General information interviewee***

- Age
- Gender
- Medical function and focus
- Years working in current function
- Years of total work experience
- Experience with video calling before VC implementation at the outpatient clinic (private or at work)
- Date and time of VC
- Type of VC appointment (follow-up, anamnesis, etcetera)
- Clinical frailty scale score (patient-specific)

***Description of appointment type***

- What does the <appointment type> look like based on the guidelines?
  - What is the goal of this <appointment type> for the condition being treated?
  - Which way of implementing this <appointment type> do you prefer?

***Evaluation perceived quality***

Starting question: What do you think video calling with frail older adult patients?

*Effectiveness*

- To what extent did you experience the VC as a fullfledged appointment?
  - Were you able to answer all questions?
  - Was a follow-up appointment necessary? If yes; how did this follow-up appointment take place (physical, video, telephone)
    - Would this follow-up appointment also have been necessary during a physical consult?
      - If yes, did you need to adjust your impression or diagnosis because information was lost during VC?
      - If no, can you explain?
- What differences have you identified between VC and regular physical appointments?
  - To what extent were you able to discuss the same subjects during VC compared to a physical appointment?

*Equivalence*

- How did the patient receive care in the desired way?
  - How were you able to provide care the way you wanted?
- During video calling, how much space was available for the patient to be able to share his/her opinion?
  - Which role did the family caregiver have during VC?
  - How was this information processed?
- How many healthcare professionals treat this patient currently?
- At the start of the interview, did you have access to all the information from these healthcare professionals regarding this patient?
- Does this contact take place with all healthcare professionals via video calling?
- What have you communicated with other healthcare professionals as a result of this conversation? How is this communicated?

*Patient centeredness*

- How does your relationship with the patient look like?
  - Is there an existing doctor-patient relationship? Did this affect the VC?
  - Did you ask for the patient’s expectations before the VC started?
  - Did you share your own expectations of the VC with the patient?
- How do you think the patient experienced this consult via video calling?
- How did the (non-verbal) communication go?
- What added value did the informal caregiver have during the video calling appointment?
- Do you think that video calling has a positive effect on the cooperation / coordination between patient, family caregiver and healthcare professional?
  - Explain why yes or no.

*Safety*

- How did you ensure during video calling that the conversation could be conducted safely?
  - How did you ensure your own privacy?
  - How did you ensure your patient’s privacy?
  - How did you ensure the family caregiver’s privacy?

*Timelines*

- Could the appointment with this patient take place in time?
  - Did the patient had to wait or did you had to wait? If yes, how long, why?
- Did no-shows occur?
  - If yes, what was done with it?

*Efficiency*

- How much time did it cost you to prepare for the VC? (changing rooms, logging in, etcetera)
- What was the duration of the VC?
  - Did this differ from e regular physical appointment? Why?
- Was there enough time during the VC to discuss everything that was desired by you in advance?
- To what extent do you know if everything you said was understood correctly?
  - What impression did the patient give you?
  - Did you get the same feedback from the patient compared to a regular physical consult?
- How did you experience the technique during the VC?
  - How did technical problems affect the appointment?
    - Which areas for improvement do you see?

***Concluding***

- Including preparation and follow-up work, a video call appointment costs me <more / same / less> time of a physical consultation. Why?
- Including preparation and follow-up work, a video call appointment costs me <more / same / less> time of a telephone consultation. Why?
  - Why? What are the differences?

***Questions for implementation***

- What are in your experience the most important advantages of VC?
- What are in your experience the most important disadvantages of VC?
- How do you see the future of VC use in geriatric care?
  - When do you see VC as a proper alternative for a regular physical consult?
  - When do you prefer which consultation type? Probes:
    - Which fase of the care process for which consultation type?
    - Which patients and patient characteristics for which consultation type?
    - Which type of healthcare professional for which consultation type?
    - Which context factors for which consultation type?
- Which software applications and which device (desktop/laptop/tablet/etcetera) do you use for VC?
  - Is it always the same? If no; why not?
  - To what extent is this software specifically meant for healthcare? If not; is it objectionable? Why?
  - Were you already familiar with this system?
    - If yes; is video calling a new addition in this system or was it already possible?
  - To what extent were you involved in the implementation of VC?
- What skills do you have to be able to video call?
  - Are these also the skills the hospital has indicated are necessary for video calling?
    - Why or why not?
  - Which skills would you further like to develop to be able to video call?
    - To what extent can this increase the use of video calling for you in the future?

**Final questions**

- What do you think that VC can add in delivering care to geriatric patients in the near future?
- Do you have additions to the subjects we discussed?

1. **Codebook**

| **Theme 1: Safety of care** | | |
| --- | --- | --- |
| **Subcategory** | | |
| **Code** | **Definition** | **Example quote** |
| Responsibility hospital | The degree to which the hospital is thought to be responsible for the safety of care during a VC appointment | **HCP5:** *I haven’t been that concerned with it. I actually assumed that it was safe if our organization chose this.* |
| (Lack of) Secure connection | The VC appointment between all users is done by a secure connection | **HCP6:** *And, well, when technology didn't work out we also sometimes did, with a child on the phone, via Whatsapp. That's not safe, a few times it did happen, I have to admit that honestly.* |
| Privacy during VC (safeguarded) | The VC appointment is held with only those who should be present. No people are present who should not be there | **HCP2:** *Of course if you really have video appointments with patients then you're just in the consulting room then the privacy on our side is no different than let’s say when you see patients physically.* |

| **Theme 2: Effectiveness of care** | | |
| --- | --- | --- |
| **Subcategory 1: Attitude of user** | | |
| **Code** | **Definition** | **Quote** |
| Acceptance of VC use | Users’ willingness to employ video consultation software use with geriatric patients. | **HCP4:** *I am now much more involved in it and I am positive about it so that helps a lot. I think we still need some more positive experiences.* |
| Attitude HCP | The settled way of thinking or feeling by healthcare providers about VC use | **HCP1:**  *It has to do with age; with wanting to change; with workload. There is a whole range of things and within our staff I am the only one together with a colleague who wants to make video calls at all. The others don't really do anything with it because, well, it's not necessary yet.*  **HCP3:** *This is at least a step in the right direction. I also think we have to make it clear to Dutch Society that this is something we are going to do. I think that is the biggest challenge because people are just not used to it.* |
| Attitude patient | The settled way of thinking or feeling by patients about VC use | **PT1:** *Well, I think anything is better than going to the hospital.*  **PT7:** *We see each other anyway. So no, I have no problem with it. As long as it goes well.* |
| Attitude family caregiver | The settled way of thinking or feeling by family caregivers about VC use | **FC3:** *And yes we had never done that before, but I thought yes I'm in, because um, you can still learn from everything even though you're at age.* |
| **Subcategory 2: Interaction during VC** | | |
| **Code** | **Definition** | **Quote** |
| Interaction | The way in which attendees experienced the interplay with each other during VC | **HCP4:** *Uhm, well you have to do something, you have to be careful that you keep talking alternately. And because there is a little delay on the line I think, yes you see the reactions of the others just a little too late.* |
| **Subcategory 3: Facilitation** | | |
| **Code** | **Definition** | **Quote** |
| Sufficient | The amount of support that users received for using VC perceived as sufficient | **HCP2:** *I think an email went out about six months ago saying that our hospital was switching to Lifesize because physical meetings were no longer possible due to the covid. I do believe there is a protocol or instructions for use where you can find out how it works and how to install it on your phone, and if things go wrong you can of course call the helpdesk. Yes, the support is there.*  **HCP4:** *In April that was all done and arranged all at once actually. Yes, including instruction on how it works, that sort of thing. They really did that quickly I think.* |
| Lack of facilitation | The amount of support that users received for using VC perceived as lacking | **HCP6:** *In the first wave, the computers that we have in the hospital in the consulting room didn't have cameras. So you had to bring your own laptop back and forth every time. That was pretty primitive.*  **HCP10**: *In the end, I worked it out reasonably well. It would be nice in itself if someone, if you have some kind of help officer who would just walk up to your poli and just demonstrate it once. That would be handy. Now you have to figure it out all by yourself and fool around and hope it works out.* |
| Communication from hospital | The way in which the hospital communicated beforehand with patients about their VC appointment | **PT5**: *I got a phone call and then I got a letter and another email on the computer giving me a code to log in. And well that actually all went fine.* |
| **Subcategory 4: Software use** | | |
| **Code** | **Definition** | **Quote** |
| Lacking skills (HCP) | The shortcoming of skills that healthcare providers have which make it difficult to use VC | **HCP1:** *I also don't yet know how to create a video contact myself with Microsoft Teams. But I expect that if I immerse myself in it for an hour or so, I'll have the skills to work with it.*  **HCP9:** *Well, indeed, start earlier with the technique. It's a bit awkward, because your clinic is obviously arranged so that you can go straight on.* |
| Lacking skills (patient/family) | The shortcoming of skills that patients or their relatives have which makes it difficult to use VC | **HCP2:** *Sometimes the informal carers are unable to download the app, for example. And sometimes the app has been downloaded and everything has been checked once, but when you want to make a video call it somehow fails.*  **FC8:** *Otherwise, she doesn't technically know how it works, doesn't know what buttons to push or how to make the connection, that's too complex.* |
| Sufficient skills (patient/family) | The sufficient amount of skills that patients and/or their relatives have which makes it easy to use VC | **HCP10:** *I found it variable and also a bit unpredictable. So it actually started pretty well. I was pleased that it did go well with this target group quite often. I thought that was a nice step. That older people still managed to find a phone or iPad and get it to work.*  **FC7:** *She could probably have done it alone, but then the nerves get the better of her and she always finds it nice to have someone around to step in if necessary.* |
| Required skills for VC | The knowledge and skills that are required for patients and healthcare providers in order to make use of VC | **HCP3:** *Well, you have to be able to operate an iPad or other device to get it to work. You have to know how to log in on an account, how to make the connection between you and the patient since you have to get the patient out of the waiting room.*  **HCP5:** *Well, you have to understand the technology. So you do have to understand how to turn it on and off and how it works. And you have to be a little more patient than usual, I think, because things can slow down a little bit because of the video calling.* |
| Technical issues | The technical issues that are perceived during video consults | **MES2:** *We have tried several times now, but is always something. Camera doesn't do it, they don't hear each other or they don't see each other.*  **FC5:** *Only the first time it went wrong. I turned the computer on at the time and she could see and hear us, but I couldn't see the doctor. And when he wanted to say something, we got a very heavy hum.* |
| **Subcategory 5: Full-fledged appointment** | | |
| **Code** | **Definition** | **Quote** |
| Value visual contact | The way in which the visual contact during VC contributes to attendees’ VC experience | **HCP10:** *But with the image attached, then you also see eh whether, for example, eh people ask for a little help with their son. Or eh people for a moment eh eh whether they become restless. Or the restlessness that you hear, whether you see that too.* |
| (Lack of) physical examination | The degree to which it was possible to perform a (physical) examination during the VC appointment | **HCP1:** *With a video call consultation, I can almost do an entire outpatient consultation, except for the physical exam.*  **HCP10**: *I did do some very limited research by having people walk or move. Yes, you can do that just fine through image calling of course. You can come quite far with that.* |
| Incomplete picture of patients | The perceived drawback of not being able to obtain a complete picture of the patients wellbeing | **HCP5:** *But after I had done it a few times I soon noticed that certain things are still difficult via video calling, despite the fact that you see the patient. You're still going to miss information that I think I need.*  **MES4:** *I think this particular patient group and their problems are so complex that video calling falls short as a medium to properly map a patient. Now we don't even always manage to properly map a patient when they are physically sitting across from you, let alone when you have to do that via video calling.* |
| Completeness of topics and questions | The degree of completeness of topics and questions asked during the VC appointment | **HCP6:** *Well maybe if we are so used to it that we also want to discuss all kinds of nasty and sensitive things like that or if you know someone really very well. But I doubt that.*  **MES4:** *We discussed all the questions, answered all the questions. We made a plan and at the end there were no more questions.* |
| **Subcategory 6: Role of family caregiver** | | |
| **Code** | **Definition** | **Quote** |
| Hetero anamnesis | The family caregiver was only present during the VC appointment to answer the hetero anamnesis questions | **HCP3:** *But so far I just find it super pleasant to do an anamnesis, particularly, a caregiver interview, a heteroanamnesis you can do just fine via video call.*  **FC9:** *Later she did ask me a few questions. We had made a list beforehand of what we wanted to know and what we had to ask.* |
| Technical support | The family caregiver was only present during the VC appointment to give technical support for the patient | **HCP2:** *Of course, there are always some elderly people who are very good at using modern equipment, but the majority of them are less competent. Yes, we then have to rely mainly on informal caregivers.* |
| Clarification | The family caregiver was only present during the VC appointment to give clarification on questions | **FC1:** *Sometimes a bit enlightening or, yes another point that my mother may have forgotten herself.* |
| General support | The family caregiver was only present during the VC appointment to give a general support to the patient | **FC7:** *I attended myself to at least make sure that it all went well, more for my mother's peace of mind. That she sat quietly and that the connection had already been made and that all she had to do was wait for the doctor to come into the picture.* |

| **Theme 3: Patient centeredness of care** | | |
| --- | --- | --- |
| **Code** | **Definition** | **Quote** |
| Responsibility patient | The way in which patients are or should be responsible for their own actions. | **HCP2:** *On the other hand, of course you don't know from where the patients call in and yes whether they have other people in that room. Yes, of course I don't know that. But I think that's also a bit of personal responsibility.* |
| Patient satisfaction | The degree to which a patient is content with the VC appointment | **PT6:** *I um, I do see it positively. But if I um, if they will go into it a bit further, or a bit deeper, I would still prefer to have a conversation at the hospital. This was a bit of general information, a general questioning, a general um yes first conversation. And I have no problem with that. But if they really eh go deeper into it then I'd rather go to the hospital.* |
| Intercourse with doctor | The way in which the doctor communicated with and supported the patient and family caregiver during the VC appointment | **PT5:** *And eh if I couldn't figure it out then eh she helped me. So she gave me a first hint, and then I remembered.* |
| Comfortness during VC | The degree in which patients and their relatives felt comfortable during the VC appointment | **PT4:** *That I don't have to go out, I don't have to drive to the hospital. Then you have to wait till it’s your turn again. So this is just easier for now.*  **PT7:** *So she also put me very much at ease.* |
| Stress level patient | The degree of physiological or psychological tension that a patient experienced during the VC appointment | **PT5:** *I feel much calmer now compared to when I have to go to the doctor.* **HCP3:** *People are often a bit tensed when they go to the doctor. Uhm and then I always say I'm going to ask you questions and then I always mention 'did you understand everything', 'are there any additional questions'. I don't have the idea that this is any different via video calling compared to the consulting room.* |
| Decision process on consultation type | The decision making process to choose between a VC and hospital appointment | **HCP13:** *Yes we have arrangements that the doctors triage, and they basically determine who comes and who doesn't.* |
| Patient self-assurance | The degree to which patients have confidence in their own abilities | **PT5:** *Well no not encumbered, but just eh that you eh a little bit afraid that it won't work out. So that's just an insecurity that I have by myself.* |
| Familiar environment | The extent to which the patient’s home environment influences the VC appointment | **HCP3:** *I do think it's the, I still think the dynamics that you see when you're video calling is different than when they're sitting in front of me. They're in their safe environment. You see much more of a sort of natural habitat.* |

| **Theme 5: Efficiency of care** | | |
| --- | --- | --- |
| **Code** | **Definition** | **Quote** |
| Multitasking | The degree in which it is possible for healthcare providers to multitask during the VC appointment | **HCP2:** *The advantage, though, is that you can see the patient and talk to them, just like I'm doing with you right now, and then at the same time if you want you can update the status in the computer.* |
| Preparation time VC | The amount of time that is needed to prepare the VC appointment | **HCP8:** *The only thing that really differed was that we really had to keep trying and be patient and helping to make this work. But once that was completed we were able to get right to the content, just like always.*  **HCP11:** *We use a specially built interface. So that in itself is fast. I just had to search again because I hadn't used it for a while.* |
| Additional work for supporting staff | The amount of additional work that medical supporting staff have in order to make VC possible | **HCP4:** *I'm not sure how laborious it is for the secretary to create the appointments. I do know that if a video call has been made for a patient once, they don't have to repeat it. So that makes a difference.*  **MES2:** *I enjoy doing it on the side, it's just a bit of a hassle to explain it and very often people are reluctant. So you have to motivate them a lot and arrange it well.* |
| Travel time | The perceived benefit of healthcare providers that patients and their relatives do not have to come to the hospital by using VC | **HCP2:** *What you can see in terms of profit is the piece of logistics that they have less. For example, an informal caregiver doesn't have to ask the whole day off in order to drive, say, to the patient and then take the patient to the hospital and then back home and then back to their own home again. So you really don't have that piece of logistical hassle anymore.* |
| Amount of workload | The amount of workload healthcare providers have due to VC use | **HCP4:** *No it doesn't add work for me. I just tell the secretary that I want a video call appointment. Uhm I do give an explanation about the video call so that is extra time, I think. But that's only a minute, it's no big deal. But video calling itself does not cost me any extra time at all.* |
| Consult duration | The duration of VC compared to TC of face-to-face consultations. | **HCP5:** *The afterwork though in the sense that the video calls never take more than an hour and an appointment at the outpatient clinic is an hour and a half. But then that's because then you also do physical examination and cognitive testing. So we reduced that time.* |
| Hospital visit after VC | Physical consultations that are required to take place after a VC appointment | **MES2:** *The physical examination is done afterwards, because it has to be done. So patients always come back for another visit.* |
| Waiting time patient/relatives | The amount of time patients/relatives have to wait on their physician to be ‘online’ to start the VC appointment | **HCP4**: *The patient has sometimes had to wait five or ten minutes before I called them. But even with that, I always warn them that I can call in the hour around the time of appointment.*  **PT3:** *We were put in the waiting room with 0 patients before us, but yes, the doctor did not come . So half an hour later my daughter said: we are going to call now.* |
| Waiting process | The waiting process that is experienced when using VC | **HCP5:** *The patient always has to wait for me because they have to log in earlier. They were also helped by the team from Video Butler. They guided people through the login process. So when I logged in, the patient was already waiting.*  **PT6:** *No, everything was told clearly to me, but the waiting part does make you a little insecure: did I do it right, do they know I'm waiting.* |

| **Theme 6: Equivalence of care** | | |
| --- | --- | --- |
| **Code** | **Definition** | **Quote** |
| Possibility to ask questions | The degree in which it is possible for patients and their relatives to ask questions during the VC appointment compared to TC and face-to-face consultations | ***I:*** *Well you told like, I was being questioned in particular, but if you had any questions by yourself did you also felt free to ask them?* **PT3:** *Yes, sure, yes.* |
| Possibility to share opinion on VC | The degree in which it is possible for patients and their relatives to share their opinion about using VC | **PT3: :** *Yes it was just an open, open contact. So like, oh we can ask that too.* |
| Value physical contact/presence | The way in which the physical contact or presence during a hospital consult contributes to a consult’s value | **MES2:** *You can just pretend to be better and wiggle away. Then a face to face contact with the doctor who sits, has a bit, anyway, has a bit more impact than this *makes hand gesture to screen*.* |

| **Boundary conditions/influences** | | |
| --- | --- | --- |
| **Code** | **Definition** | **Quote** |
| Boundary condition | The requirements that must be met in order to use VC | **HCP6:** *So, people do need to possess the cognitive skills to understand that it is indeed you on that screen. Yes.* |
| Information sharing | The perceived benefit of receiving and sharing information while using VC | **HCP4:** *It's nice if you can share that screen. I think that when you see someone at the outpatient clinic it's often very good that you can literally show them the results. It's nice for the patient to see what happened. That would be a nice addition.* |
| Doctor-patient relationship | VC use that is experienced as effective due to the presence of an existing doctor-patient relationship | **MES3:** *I do think for a first consultation it's nice to see a doctor, to have a little bit of a personal touch. But for follow-up appointments, I wouldn't think it matters that much whether it's via video calling or whether you have physical appointments.*  **PT8**: *No, I don't think that's so important, no. I don't really recognize the doctor but it has to be done anyway, I think to myself. I don't really mind.* |
